# Views of students with disabilities on how institutional support shapes their experiences

**DOI:** 10.4102/ajod.v14i0.1553

**Published:** 2025-05-29

**Authors:** Emeldah C. Munjanja, Eleanor A. Hendricks

**Affiliations:** 1 Department of Social Work, Faculty of Social Sciences and Humanities, University of Fort Hare, Alice, South Africa; 2 Department of Social Work, Faculty of Humanities, University of Limpopo, Mankweng, South Africa

**Keywords:** students with disabilities, higher education, institutional support systems, disability unit, student counselling unit

## Abstract

**Background:**

Students with disabilities in higher education continue to face significant challenges, including infrastructural barriers and stigma, which hinder their full participation. Although disability and student counselling units have been established, their effectiveness may be questionable.

**Objectives:**

Our study explored the perceptions of students with disabilities (SWDs) at selected higher education institutions (HEIs) in Raymond Mhlaba, Eastern Cape, regarding the effectiveness of institutional support systems in shaping their academic experiences.

**Method:**

A qualitative approach was employed, using semi-structured interviews to gather data from 15 SWDs at selected institutions. Data were thematically analysed.

**Results:**

Findings revealed that institutional support systems played a crucial role in assisting students by providing counselling, wheelchair-accessible residences, food parcels and motorised wheelchairs. However, some participants noted that some services, such as assistive technology and academic accommodations, were not well advertised to students by Disability Offices. Consequently, only students who disclosed their disabilities accessed these services, while those who did not disclose remained unaware and unsupported.

**Conclusion:**

Despite the crucial role of institutional support systems, there is a significant gap between students’ awareness of and ability to access or use the services. Strengthening communication and encouraging students to disclose their disabilities through efforts by administrators, disability services and faculty staff could improve access to support.

**Contribution:**

Our study contributes to the understanding of how HEIs can create inclusive environments that promote academic and social integration for SWDs.

## Introduction

The global discourse on inclusivity and accessibility in education has gained significant momentum in recent decades. More recently, there has been a determined effort globally to overcome the challenges faced by students with disabilities (SWDs) in higher education (HE) by establishing disability units (DUs) and student counselling units (SCUs) adapted to the requirements of SWDs, and South Africa is no exception (Mbuvha 2019). However, the efficacy of these support systems in shaping the experiences of SWDs is a significant problem, as the support systems in place are not effective enough to positively impact these students’ experiences (Mbuvha 2019). Despite the efforts of institutional support systems, challenges persist in ensuring equal access and opportunities for SWDs in higher education institutions (HEIs) in South Africa (Munjanja & Hendricks 2024). Limited funds, inadequate infrastructure and a lack of understanding about disability concerns are still important impediments that must be addressed (Mutanga 2017).

According to Mbuvha (2019), the DUs and SCUs seek to help SWDs get access to and participate in university life. This includes implementing reasonable accommodations and supporting SWDs to ensure their full involvement in academic, social and extracurricular activities and equal opportunities as their peers, which is in line with the United Nations Convention on the Rights of Persons with Disabilities (UNCRPD 2006), which was ratified in South Africa in 2007. Students with disabilities who may require support and accommodations include students who have hearing disabilities, visual disabilities, physical disabilities, health conditions (such as chronic illnesses), specific learning disabilities, intellectual disabilities, developmental disabilities or psychosocial disabilities.

Our study explores how effectively institutional support systems foster full participation and inclusion of SWDs, ensuring that SWDs have equal opportunities to engage in all aspects of university life, including academics, social activities and support services, without facing barriers or discrimination. Through highlighting the strengths, weaknesses and areas for improvement within these systems, our study provides actionable insights that may help to guide policy changes and resource allocation, fostering a more inclusive and supportive environment for SWDs in HEIs (Munjanja & Hendricks 2024).

South African HEIs have set up DUs and SCUs to assist SWDs in academic pursuits (Mantsha 2016). These DUs are vital in providing services, including offering study materials in accessible formats such as Braille, allowing additional time for examinations and arranging for sign language interpreters (Nsanja Gunda 2015). The DUs and SCUs offer student accommodations, emotional support and counselling services, respectively, tailored to the needs of SWDs. Grigal et al. (2018) noted that transitioning to HE can be challenging and that SCUs provide a safe space for SWDs to voice their concerns and difficulties. Therefore, these units strive to create an inclusive and accessible atmosphere that promotes both academic and personal success for SWDs.

On the contrary, Sims (2022) stated that DUs are not independent institutions; instead, they are merged into other departments, such as student affairs or counselling, which can restrict their effectiveness in supporting SWDs as they often lack the autonomy to offer specialised services to SWDs. However, it is essential to recognise that some SWDs may choose not to seek counselling services. Hartrey, Denieffe and Wells (2017) identify several reasons for this hesitation, such as a preference for privacy, concerns and personal beliefs about the stigma surrounding mental health counselling or psychological support.

The effectiveness of the institutional support systems at selected HEIs in Raymond Mhlaba, Eastern Cape, South Africa, in shaping the experiences of SWDs raises significant apprehensions among SWDs themselves, as well as HE administrators, educators and policymakers. This is because SWDs are still facing challenges in HEIs in South Africa, regardless of the support they receive from their institutional support systems (Munjanja & Hendricks 2024). Mutanga (2017) highlights many impediments in HE that contribute to this predicament, including unfavourable attitudes about disability, gaps in academic courses, physical difficulties (inaccessible buildings and inadequate transportation), a lack of appropriate support services, such as tutors and personal assistants, and unequal distribution of resources, where institutions may prioritise students without disabilities, leaving SWDs inadequately resourced. Gaps arise in academic courses as there is a lack of specialised or adapted educational programmes for SWDs, which affects their learning opportunities. As a result of these impediments, many SWDs drop out of university, and others cannot complete their tertiary education (Mutanga 2017).

According to the 2021 South African statistics on the causes of student absenteeism from university, the leading factors were classified as follows: health concerns and impairments at 22.7%, poor academic performance at 21.2% and inability to pay tuition fees at 19.6% (Stats SA 2021). These figures indicate that health issues and disabilities are a central contributor to university dropout rates in South Africa. These data suggest that the efficacy of institutional support systems may be questionable, although there are notable contributions.

Our study aimed to explore how institutional support systems shape the experiences of SWDs at selected HEIs in Raymond Mhlaba, Eastern Cape, South Africa.

## Research methods and design

### Study design

A qualitative study with an exploratory research design underpinned by an interpretivist paradigm was conducted (Dannels 2018) at two South African HEIs such as University of Fort Hare (UFH) and Lovedale College TVET (LC) in Raymond Mhlaba Local Municipality, Eastern Cape.

### Study population and sampling strategy

We used purposive and snowball sampling methods to find and recruit potential participants (Parker, Scott & Geddes 2019). Fifteen participants aged 20–56 years were purposefully sampled. The first author initially approached the DU of the University of Fort Hare and the Student Support Office of Lovedale College TVET (LC), which serves as DU and SCU. Lovedale College does not have an independent DU and SCU; hence, they use the Student Support Office to assist SWDs and those without disabilities in requesting access to SWDs listed in their registers who were willing to participate in our study. The DU communicated the purpose and goals of our study to potential participants and provided them with the first author’s contact information. Interested students could then contact the first author via email or phone to express their willingness to participate. Through this iterative snowball sampling process, individuals who were SWDs studying at the University of Fort Hare and Lovedale College, as well as staff at the DU, SCU and Student Support Office of these two institutions, were selected (Munjanja & Hendricks 2024).

At Lovedale College, seven participants volunteered to participate, and eight volunteered at the University of Fort Hare. The recruitment process took one month. It reached saturation after 15 participants, marking the conclusion of the recruitment process.

We chose the University of Fort Hare and Lovedale College TVET because they offer diverse study environments, from well-established universities to vocational and technical education institutions, ensuring comprehensive insights into support systems across various educational contexts.

### Data collection

Face-to-face and online semi-structured interviews were conducted to collect data from participants. Semi-structured interviews allowed participants to express their perspectives in depth and allowed the first author to obtain more information from the participants by asking follow-up questions (Kallio et al. 2016). Interviews were audio recorded. Recording the participants’ responses allowed for additional information beyond what was written down during the interview and facilitated later transcription. A research assistant was responsible for recording face-to-face interviews and writing notes. Because of impending examinations, some participants opted for virtual platforms like Zoom and WhatsApp for interviews. Two students were interviewed via Zoom meetings, four via WhatsApp calls, and nine participants were interviewed face-to-face. The participants were interviewed in English; each interview lasted for approximately 30 min, and the interviews were held in private settings to ensure confidentiality (Munjanja & Hendricks 2024).

### Data analysis

The six steps of thematic data analysis by Braun and Clarke (2006) were used to analyse data. Data were first transcribed, which involved attentive listening to recorded interview sessions and accurately typing the content into textual data. The first author and a research assistant created the first codes by categorising data related to the participants’ experiences, perceptions and interactions with institutional support systems, and comparable codes were compiled into themes for further analysis. Following that, the findings were examined in relation to these coded themes. The concepts and conclusions were communicated through a narrative with supporting data quotes. To guarantee the quality and trustworthiness of the data, three critical criteria were considered such as credibility, transferability and dependability (Nowell et al. 2017). Following suggestions by Anney (2014), credibility was ensured through ongoing participant engagement to validate findings and regular debriefing sessions between the first author and supervisor to address challenges. Transferability was ensured by refining our study’s procedures and findings through multiple phases, including member checks, to achieve a detailed and comprehensive description. Dependability was ensured by the researchers diligently recording decisions and activities throughout our study, keeping comprehensive records to ensure a clear audit trail (Anney 2014, Munjanja & Hendricks 2024).

### Ethical considerations

Ethical clearance to conduct this study was obtained from the University of Fort Hare Research Ethics Committee (UREC) (No. Rec-270710-028-RA). Participants signed informed consent.

## Results

Our study explored how institutional support systems influence the experiences of SWDs in HE. Five key themes emerged: (1) students are aware of their institutional support systems, but are unsure of the services they provide; (2) services are available to students who have disclosed their disabilities; without disclosure, institutional support systems may not be able to reach students; (3) structural barriers to inclusion: the urgent need for university action and policy reform; (4) there is insufficient collaboration between the DU, university departments and external support systems; and (5) the lack of independent DUs hinders students from disclosing their disability and accessing services freely (Munjanja & Hendricks 2024).

### Characteristics of the sample

Fifteen SWDs participated – nine men and six women. Among them, 13 had physical disabilities, 1 had a psychosocial disability, and 1 had a partial vision impairment. The group comprised 4 postgraduate and 11 undergraduate students from different academic disciplines. The diverse backgrounds of the participants played a crucial role in shaping our findings, offering a comprehensive understanding of the experiences of SWDs in HE. Representing male and female students enabled a gender-sensitive exploration of institutional support systems.

#### Theme 1: Students are aware of their institutional support systems, but are unsure of the services they provide

Twelve students were aware of their institutional support systems and informed by staff after first-year registration. Two learned about them later through university staff and peers, while one discovered them after developing a disability. However, 7 of the 12 students remained uncertain about the services provided. A fourth-year Human Settlement student recalled their experience during registration in 2019 and shared:

‘The staff member who saw me on crutches during my first year at Sports Complex during registration informed me about the DU and the SCU. I have never visited these units because I am unsure of the services they offer.’ (Student 1, UFH, 20 years, male)

Similarly, Student 7, who has a psychosocial disability and physical disability, expressed:

‘During my first-year application, I disclosed my disability. The Disability Unit later contacted me and explained their services. Even though they explained their services to me, I am still unsure what help I can get from them.’ (Student 7, UFH, 23 years, female)

Student 15, who also has a physical disability, stated:

‘During my first-year orientation, DU and SCU staff explained their services and office locations. Though it was clear, I had unanswered questions but was too shy to ask.’ (Student 15, LC, 27 years, male)

While some students learned about support systems early, others spent years at their institutions unaware of these services. Student 13, a third-year student with a physical disability, shared:

‘I missed orientation in my first year and never heard of the DU and SCU. In my second year, my residence warden informed me, and I later registered with the DU.’ (Student 13, LC, 25 years, male)

Student 8, a fourth-year student of Bachelor of Social Sciences, only learned about her institutional support systems after developing a disability. She said:

‘Following a bone fracture and thumb amputation, her doctor provided a letter outlining her limitations. After giving it to her lecturer, she was referred to the DU.’ (Student 8, UFH, 24 years, female)

Unlike Students 13 and 8, Student 14, who has a physical disability and was a second-year student of Bachelor of Office Administration, learned about support services from a fellow student:

‘Another student with a disability mentioned the DU when we were having a general conservation. That is when I got to know.’ (Student 14, LC, 29 years, male)

#### Theme 2: Services are available to students who have disclosed their disabilities; without disclosure, institutional support systems may not be able to reach students

Students reported receiving services from their institutional support systems only after registering with their DUs. Also, the students who got to know of their DU later did not receive services in the early years of their studies until they disclosed their disabilities to their DUs. Student 1 stated:

‘I disclosed my disability at the DU when I was a first-year during registration, and the DU has been supporting me.’ (Student 1, UFH, 20 years, male)

Student 12 similarly shared:

‘During orientation, I registered my name with the Student Support Office, and I go there every time to print my assignments.’ (Student 12, LC, 20 years, female)

Student 2, on the other hand, said:

‘I got to know of the DU in my third year. I was surprised the day I registered my name and was informed about the services offered by our DU.’ (Student 2, UFH, 28 years, male)

#### Theme 3: Structural barriers to inclusion: The urgent need for university action and policy reform

Participants emphasised the ongoing structural barriers that hinder their full inclusion in HE. They highlighted that while some accommodations exist, they fail to address the broader systemic challenges. Issues such as inaccessible infrastructure and outdated policies remain significant obstacles, requiring urgent intervention and comprehensive policy reform to ensure meaningful inclusion.

Many students faced significant barriers related to accommodation and mobility on campus. Student 5, a fourth-year student and a wheelchair user, shared:

‘The DU wrote a letter to the residence office on my behalf, and I was given a single room with an ensuite bathroom. However, I cannot visit my friend on the upper floor there are no lifts. Our residences and classrooms should have lifts to move freely.’ (Student 5, UFH, 24 years, female)

Similarly, Student 2 stated:

‘The DU arranged transport, but there are delays even when I book in advance. I wish I could move around like my peers. The institution should build accessible residences closer to classrooms.’ (Student 2, UFH, 28 years, male)

Student 15 also noted:

‘Although the institution provided a motorised wheelchair, the lack of sufficient ramps on campus and the stone-covered ground still makes it difficult for me to navigate on my own. Ramps are very important; we should have more ramps in our institution.’ (Student 15, LC, 27 years, male)

Beyond physical barriers, students also faced social challenges. Student 9 shared their experience with stigma and how counselling helped:

‘When I arrived, I faced stigma from other students. I went to the SCU. After six sessions, I learned to accept myself, but the stigma continues. The university should raise awareness to educate students about disabilities.’ (Student 9, UFH, 30 years, female)

Participants expressed that they acknowledged that their institutions were trying to help them but were still facing challenges. For instance, although Student 5 received a single room, their movement within the residence remained restricted because of the lack of lifts. Similarly, Student 15 was provided with a motorised wheelchair, while the campus infrastructure, such as inadequate ramps and rough terrain, still limited independent mobility. Student 2 had access to institutional transport, but delays and the distance of disability-friendly residences from classrooms hindered their full participation. Student 9 faced initial stigmatisation and sought support from the SCU. While the counselling helped the students accept themselves and cope with stigma, the ongoing challenge of societal acceptance remained unresolved. The student suggested that the unit’s efforts could be more effective if they included broader awareness and education initiatives to address the root cause of stigmatisation of persons with disabilities.

#### Theme 4: There is insufficient collaboration between the disability unit, university departments, and external support systems

Participants expressed disappointment when they needed assistance from their DUs that involved other university departments and external support systems like financial bursaries, university faculty and the administration office. The following are the verbatim expressions of the participants:

Student 14 shared that the Student Support Office was always responsive whenever they sought assistance and expressed:

‘They respond quickly, but faculties and administration often delay. Other departments should be educated to assist us on time.’ (Student 14, LC, 29 years, male)

Similarly, Student 7 expressed frustration with the system when encountering difficulties with their NSFAS (National Student Financial Aid Scheme) meal allowance and explained:

‘As an NSFAS beneficiary, I faced meal allowance delays. The DU referred me to the Bursary Office, but both said it was beyond their control. I felt disappointed and stuck, losing trust in the DU.’ (Student 7, UFH, 23 years, female)

Student 12 also recounted a similar struggle in accessing financial assistance and shared:

‘I went to the student support office when I did not receive my meal allowance for October. They referred me to finance, and I was told to follow up with NSFAS. NSFAS said I was not in the system, even though this is my 4th year receiving allowances.’ (Student 12, LC, 20 years, female)

#### Theme 5: The lack of independent disability units hinders students from disclosing their disability and accessing services freely

Participants from one of the selected institutions reported that their DU is part of the Student Support Office, which serves all students. They felt this setup discouraged them from seeking help and disclosing their disability. One participant shared:

‘I first visited the DU when registering, but the office is for every student on campus. This makes me hesitant to go, especially with my invisible disability, as I have to disclose it to them repeatedly. They should have an office for SWDs only.’ (Student 13, LC, 25 years, male).

Some SWDs indicated that they concealed their disabilities because the office responsible for supporting them lacked privacy, as it also served students without disabilities. Student 10 shared:

‘I have friends who are in their third year, and they have invisible disabilities. They vowed not to step foot in that office; they feel there is no privacy.’ (Student 10, LC, 22 years, male)

## Discussion

While SWDs may be aware of the existence of DU at their institutions, there is a clear gap in their understanding of the full scope of services offered. This issue is critical because many students indicated that the DU was their first point of contact for assistance, but many remained unsure about the exact nature of support available. This is like Kim and Crowley’s (2021) argument that HEIs often fail to effectively communicate the full range of available support services, leading to limited student awareness. This lack of outreach is especially evident when students miss crucial orientation events or fail to disclose their disabilities during registration. Consequently, students may navigate their academic experiences with insufficient institutional support, fostering a sense of self-reliance because of the absence of clear communication (Aithal & Aithal 2023). To address this, institutions should prioritise proactive outreach initiatives and awareness campaigns to ensure that all students, especially new enrollees, are well-informed about the support services available to them (Munjanja & Hendricks 2024).

A key issue highlighted in our study is the link between disability disclosure and access to institutional support services. Higher education institutions are legally obligated to provide support, such as alternative learning materials and modified assessment methods, once students disclose their disabilities (Cinarbas & Hos 2022). However, to access these, students must first disclose their disability and engage with the DU, which may also include benefits like stipends specifically for SWDs (Brewer, Urwin & Witham 2023). While students who disclose their disabilities can access specialised services, including extended exam time and assistive technologies, those who do not disclose are often excluded from these provisions (Becker & Palladino 2016).

The reluctance to disclose disabilities is rooted in several factors, such as the fear of stigma and negative labelling by lecturers and peers (Grimes et al. 2019). The findings in Grimes et al.’s (2019) study had similar findings, where some students chose not to disclose their disability out of fear of being stigmatised by both their peers and lecturers. Furthermore, as observed by Grimes et al. (2019), some SWDs may not consider themselves as having a disability or may prefer to navigate their education without additional support. However, this hesitancy to disclose significantly limits students’ access to vital institutional support, reinforcing the critical role of disclosure in ensuring that SWDs can access the accommodations necessary for their academic success.

Despite the presence of institutional support systems, the assistance provided to SWDs often lacks sustainability and fails to address the systemic barriers that hinder their full participation in HE (Mbuvha 2019). Our participants highlighted infrastructural barriers and stigma as barriers to their learning. While some institutions implement disability support measures, these are often reactive and fragmented rather than part of a strategic, long-term commitment to inclusion (Mutanga 2017). Therefore, universities should adopt a twin-track approach, integrating mainstream support structures that accommodate all students while ensuring targeted interventions that address the specific needs of SWDs (United Nations 2012). This means embedding considerations of disability, gender and other intersectional factors into all policies, planning and programme implementations, ensuring that institutional responses are not merely compensatory but transformative. As Vincent and Chiwandire (2017) argue, SWDs will continue to face challenges in equitably accessing education without addressing the structural barriers perpetuating exclusion (Munjanja & Hendricks, 2024).

To achieve meaningful inclusion, universities must go beyond providing temporary accommodations and work towards systemic change that ensures that SWDs have equal access to education and opportunities for success (Mbuvha 2019). This requires the integration of disability-specific interventions within mainstream services, ensuring that students receive support without feeling isolated or marginalised. Institutions must actively address stigma and discrimination within the student body and in institutional culture by fostering environments that prioritise accessibility, equity and inclusivity (Vincent & Chiwandire 2017).

Another important issue is the lack of coordination and communication between the DU and other university departments. Although DUs are intermediaries between SWDs and various university functions, this role was not always executed efficiently. For example, some students noted delays in accommodations because of poor communication between the faculty staff, the DU and the facilities’ management teams. This issue is like Kilpatrick et al. (2016), who reported that students and staff identified poor communication as a critical barrier to providing timely and effective support to SWDs. The failure to ensure that faculty staff were adequately informed about the necessary accommodations further exacerbated the challenges faced by SWDs, particularly when academic materials were inaccessible or when classroom settings were not adjusted to meet the students’ needs (Kilpatrick et al. 2016).

Moreover, the collaboration between DUs and external support systems, such as government funding programmes like the National Student Financial Aid Scheme (NSFAS), remains insufficient. Vincent and Chiwandire (2019) noted that NSFAS introduced a student loan plan in 1996 to assist underprivileged yet capable students in pursuing HE. The NSFAS programme aimed to provide opportunities for education and post-secondary training while offering additional support to help students overcome the challenges of disability (Sokhweba 2022). However, many students reported difficulties in accessing NSFAS funding and receiving timely support, which reflects broader issues of insufficient resources and poor communication, ultimately hindering the full inclusion of SWDs in academic life. This highlights the need for government intervention to address these barriers, specifically by ensuring that under-resourced students receive mainstreamed support through Track 1 and SWDs are provided with additional assistive devices and targeted interventions through Track 2 of the twin-track approach (Dhanda 2020). Higher education institutions must strengthen their partnerships with external agencies and improve internal communication to address students’ needs comprehensively and promptly.

The absence of independent DUs at some institutions remained a significant barrier to the students’ ability to disclose their disabilities and access the support services they needed. The DU is a fully equipped entity that provides financial and human resources to SWDs (Chiwandire 2020). Many students prefer separate units as they offer a more discrete and supportive environment, reducing the perceived shame or judgement associated with disclosing a disability. Our findings are similar to those of Zhang (2024), who argued that independent DUs are critical in creating an environment where students feel confident and empowered to disclose their disabilities. Without a dedicated office, students may feel that their needs are secondary to the broader objectives of the other departments, thereby limiting their access to tailored support services (Zhang 2024). The lack of an independent DU also impeded the visibility of disability services, making it harder for students to navigate the system and understand the full range of support available. Munjanja and Hendricks (2024) recommended that institutions must prioritise the establishment of independent DUs that can serve as central hubs for disability-related services, ensuring confidentiality and accessibility.

De Cesarei (2015) added that establishing independent DUs would help create a more supportive and inclusive environment for SWDs, facilitating their academic success and social integration. This implies that the setting in which students decide to reveal their disability is important (De Cesarei 2015). Thus, institutional support systems should create a secure and independent environment and a trusting culture to encourage disability disclosure (Potts 2017). Many participants expressed a reluctance to engage with integrated services because of past experiences of discrimination, exclusion or being perceived as burdensome by their peers and staff. This preference for separate spaces may, in part, reflect the deep impact of stigma and self-stigma, where students internalise societal attitudes and seek to distance themselves from able-bodied peers to avoid further marginalisation (Mutanga 2017).

Additionally, some students may have developed a sense of mistrust towards mainstream institutional services, perceiving them as inadequate in addressing their specific needs (Nwangwu 2021). This suggests that beyond structural challenges, the emotional and psychological dimensions of disability experiences play a crucial role in shaping students’ preferences for independent support structures. While independent DUs provide a safe and affirming environment, this raises critical questions about the effectiveness of broader institutional inclusivity efforts. Makuwira (2022) added that universities must consider how to balance dedicated disability support with mainstreaming efforts in a way that both mitigate stigma and foster a truly inclusive campus culture. Independent DUs create an atmosphere where SWDs feel their needs are validated, fostering trust and openness. This approach supports the idea of Track 2 of the twin-track approach, which targets the specific needs of SWDs while ensuring their inclusion in the broader academic system (United Nations 2012).

The potential for mainstreaming with dedicated support for disability-specific needs, as outlined by the twin-track approach, presents an interesting solution. The United Nations (2012) noted that Track 1 of the twin-track approach focuses on mainstreaming support for all students, including those under-resourced; meanwhile, Track 2 provides targeted interventions, including assistive devices and tailored services for SWDs. This dual approach ensures that SWDs are fully included in their academic pursuits while receiving the specific support they require (Nwangwu 2021). The challenge, however, lies in the practical implementation of the twin-track approach, especially in contexts where resources are limited and communication between departments is weak. However, when DUs are isolated, it may limit the potential for SWDs to engage fully in mainstream activities. Therefore, institutions should aim to strike a balance between independent DUs and integrated support, ensuring that both specialised resources and inclusive mainstream systems work together to offer a holistic approach to student support (Dhanda 2020; Munjanja & Hendricks 2024).

### Limitations

Although we sought to collect extensive participant data to achieve our objectives, there were some limitations. Interviews were conducted both face-to-face and virtually. However, virtual interviews posed challenges, particularly with network unreliability. The interruptions caused by poor network connectivity led to incomplete responses, resulting in rescheduled interviews, which could be viewed as a limitation because of potential delays in data collection and inconsistent communication. Also, many participants were students with physical disabilities, with one participant having a psychosocial disability and one with partial vision disability. This limited range of disability types may not fully capture the diverse experiences of SWDs, such as sensory, intellectual or mental health disabilities. Despite these obstacles, data collection was ultimately successful.

### Recommendations

Recommendations for the study are as follows:

- Future research should include a more diverse group of students with different disabilities to better understand the impact of institutional support systems across various experiences.
- Based on the students’ perspectives, we suggest that support systems create and share a comprehensive guide on available services, in print and online, and promote it via orientation, emails, social media and campus posters.
- Support systems should develop long-term programmes, including ongoing counselling, skill-building workshops, mentorship and tailored academic accommodations, to address students’ core challenges.
- HEIs are urged to build independent DUs to create a safe space for students to disclose their disability. However, where resources do not currently allow for this or where universities aim to adopt a mainstreaming approach, institutions should implement measures to address key barriers, particularly stigma. This can include fostering a culture of inclusivity through awareness campaigns, staff training and clear policies that promote confidentiality and non-discrimination.

## Conclusion

Institutional support systems foster inclusivity and accessibility in HE for SWDs. While these systems provide essential services such as academic accommodations, emotional support and advocacy, their effectiveness is often hindered by structural limitations, resource constraints and a lack of independent DUs. Our study highlights the critical need for universities to strengthen these support mechanisms to ensure that they are accessible and sustainable. A key barrier to effective support is the requirement for students to disclose their disabilities to access services. Many students choose not to disclose their disability due to stigma, a lack of trust or concerns about confidentiality, ultimately excluding them from the needed assistance. Additionally, where DUs are integrated within other departments, their visibility and effectiveness are compromised, making it harder for students to access timely and specialised support.

Moving forward, universities must prioritise strategies that promote both mainstreamed and targeted support, aligning with the twin-track approach. Establishing independent DUs, improving resource allocation and fostering a culture of inclusivity are essential steps to ensuring that SWDs receive the support necessary for their academic success.
